# Horizontal-scanning attenuated total reflection terahertz imaging for biological tissues

**DOI:** 10.1117/1.NPh.7.2.025005

**Published:** 2020-06-13

**Authors:** Limin Wu, Degang Xu, Yuye Wang, Yingying Zhang, Hanjie Wang, Bin Liao, Sheng Gong, Tunan Chen, Nan Wu, Hua Feng, Jianquan Yao

**Affiliations:** a Tianjin University, Institute of Laser and Optoelectronics, School of Precision Instruments and Optoelectronic Engineering, Tianjin, China; b Tianjin University, Key Laboratory of Optoelectronics Information Technology (Ministry of Education), Tianjin, China; c Tianjin University, School of Life Sciences, Tianjin Engineering Center of Micro-Nano Biomaterials and Detection-Treatment Technology, Tianjin, China; d Third Military Medical University (Army Medical University), Southwest Hospital, Department of Neurosurgery and Key Laboratory of Neurotrauma, Chongqing, China

**Keywords:** terahertz wave, terahertz imaging, attenuated total reflection, brain tumor

## Abstract

**Significance:** Terahertz wave is a potential tool for biological tissues due to its noninvasiveness and high sensitivity to water. Attenuated total reflection (ATR) with the characteristics of high sensitivity and nondestruction has been applied for THz imaging.

**Aim:** We aim to develop an imaging methodology to facilitate practical application of THz ATR imaging.

**Approach:** We have demonstrated a horizontally scanning THz continuous wave ATR imaging system. The effective imaging area was as large as the prism imaging surface by optimizing the ATR prism, and the influence of secondary reflection can be well avoided. By taking the image resolution and stability of this system into consideration, the incident angle α to the prism bottom was chosen to be 30 deg.

**Results:** The image resolution of this system can be up to 400 and 450  μm in horizontal and vertical directions, respectively. Furthermore, U87-glioma regions of mice brain tissues with different sizes and C6-glioma regions of rat brain tissues with relatively large size can be differentiated clearly from normal brain tissues by this imaging system. The volume and location of the tumor region shown in the THz images are similar to those visualized macroscopically in the corresponding visual and H&E-stained images.

**Conclusion:** We indicate terahertz horizontal-scanning ATR imaging technique with large effective imaging area, and high resolution could be used as an alternative method for label-free and high-sensitivity imaging of biological tissues.

## 1 Introduction

Terahertz (THz) imaging techniques have attracted great attentions in biological imaging, based on its high sensitivity to water, noninvasiveness, and nonionizing characteristics.^1^ THz-wave application in biomedicine has been widely used for both distinguishing tissue types^2^^,^^3^ and lesion recognition.^4^^,^^5^ At present, THz imaging systems mainly adopt transmission and reflection modes. However, biological tissue generally has a high water content, especially *in-vivo* and fresh *ex-vivo* biological samples. For high absorption sampling, THz-wave transmission mode needs sample fabrications to get thin-sliced solids.^6^ This would cause sample moisture loss and prolong experiment time. Although reflection mode can ensure sample integrity, it has lower accuracy because of diffuse reflection.^7^ Especially, the reset of a gold mirror for obtaining the reference signal increases the measurement error because the biological tissue is usually soft and closely clings to the imaging window, whereas an air gap would exist between the gold mirror and imaging window. To our delight, attenuated total reflection (ATR) mode can provide information on the interaction between the sample and evanescent wave traveling along a prism surface. ATR mode can ensure sample integrity and has the characteristic of high sensitivity. Moreover, it is noteworthy that the reference signal measurement for ATR mode can be measured by removing the sample.^8^

THz ATR spectroscopy has experienced a long-term development and has many applications in biomedical studies, such as detection of live cells, lesion cell recognition, and the determination of many other biological samples.^9^^–^^11^ However, THz ATR imaging has just been emerging. Gerasimov et al.^12^ obtained a real-time THz ATR imaging of an alcohol droplet into water with free-electron laser and microbolometer array detector. Sample details in this test were not shown because of the low imaging resolution of system. Wojdyla and Gallot^13^ deposited samples on a patch and achieved a THz ATR image of a frog sciatic axone by directly scanning this patch moving on the top of a fixed prism based on THz time-domain spectrometer. These two components of this system must be in close contact, which may cause scratches and emergence of evanescent wave in their gap. Compared to the pulsed THz imaging system, a continuous wave (CW) imaging system can afford higher output power and relatively fast scanning time.^14^ In addition, the scanning time of CW imaging system can be further improved using an array detector, but this kind of detector has lower resolution.^15^ Thus, point scanning is more suitable for the THz imaging system at present. Recently, Liu et al. in our group developed and optimized a vertically scanning CW THz-ATR imaging system.^16^^,^^17^ The THz-ATR images of water droplet and solid agar have been realized using p-polarized THz-wave with enhanced image contrast. However, this system still has the disadvantages of secondary reflection inside the prism and small imaging area. There is still an urgent demand for developing new imaging methodology to facilitate practical application of THz ATR imaging.

In this paper, we have demonstrated a horizontally scanning CW THz ATR imaging system. This scanning method can realize a large effective imaging area as well as avoid the influence of secondary reflection on imaging results by optimizing prism design. The principle and feasibility of this system are presented theoretically and experimentally, showing good agreement. By taking the image resolution and stability of this system into consideration, the THz wave incident angle α to the prism bottom in air was recommended as 30 deg. Furthermore, the imaging capabilities of this method were investigated on glioma samples. C6-glioma regions of rat brain tissues and U87-glioma regions of mice brain tissues can all be differentiated clearly from normal tissues of the corresponding samples using THz horizontal-scanning ATR imaging system. The results of THz imaging are similar to those visualized macroscopically in the corresponding visual and hematoxylin and eosin (H&E) staining images.

## 2 Methodology

### 2.1 Experimental Setup

**Fig. 1:**
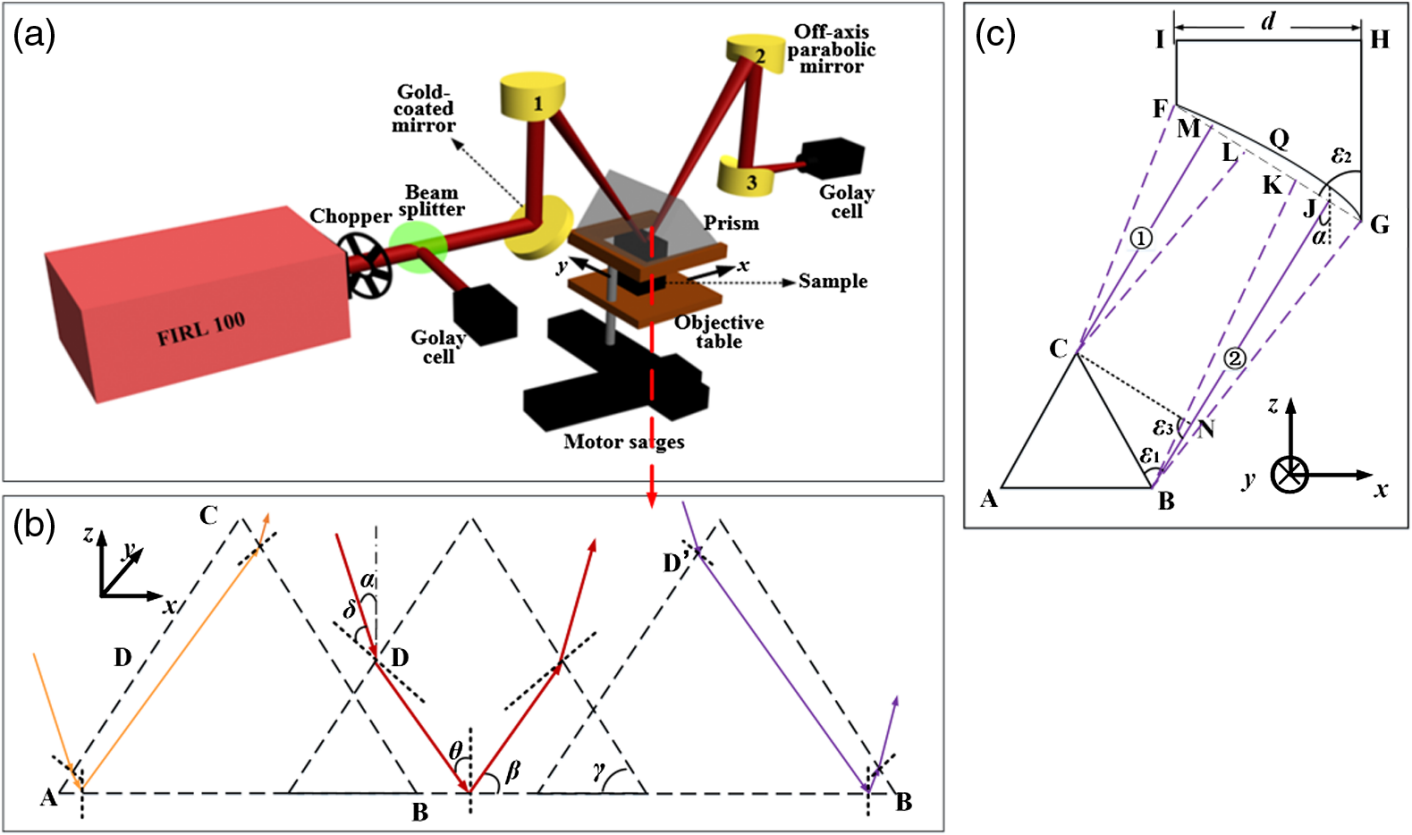
(a) Experimental setup. (b) Schematic diagram of the scanning principle at x-axis. (c) Schematic diagram of off-axis parabolic mirror receiving THz signal.

The schematic diagram of the experimental system is shown in Fig. 1(a). An optically pumped THz gas laser (FIRL100, Edinburgh Instruments Ltd.) with tunable continuous THz-wave was used in this study. The frequency of 2.52 THz was chosen and the maximum output power was 150 mW. The THz-wave was separated into two beams by wire-grid beam splitter (Micromesh Instruments, Inc.); one beam served as a signal and the other was used as a reference. The reference light was received directly by the Golay cell detector (GC-1P, Tydex Ltd.). The signal light was reflected and focused by one gold-coated flat mirrors and the off-axis parabolic reflector labeled by number 1, respectively. The off-axis angle of parabolic reflector 1 was 30 deg. Then, the focused signal light was incident onto an isosceles triangle-shaped silicon (n=3.42 at 2.52 THz) prism, whose size was $34.8\times 34.8\times 20\;{\mathrm{mm}}^{3}$ with base angle of 49 deg. Considering the refractive index of biological samples is usually smaller than that of water (n=2.05 at 2.52 THz), the incident angle of 43.5 deg at the prism bottom is always certain to be larger than the theoretical critical angle, which is 34.8 deg for distilled water. The prism and objective table were both mounted on a two-dimensional (2-D) motor stage (Sigma Koki Co., Ltd.). The focus of the signal light was located at the bottom of prism. Sample was fixed on the objective table, and the upper surface of sample was adjusted to closely contact with the bottom surface of prism for THz imaging. Considering the sample and prism were scanned simultaneously, the contact of the prism with sample can be well maintained during the imaging process. The scanning step was set as 200  μm in this experiment, and the scanning speed was about 10  pixels/s. The signal light with sample information was collected and focused by the off-axis parabolic reflector labeled by numbers 2 and 3, respectively. The focal length of parabolic reflector labeled by numbers 1, 2, and 3 was 2, 2, and 4 in., corresponding to the F-number of 1, 1, and 2, respectively. The off-axis angle of parabolic reflector labeled by numbers 2 and 3 was 30 deg and 90 deg, respectively. Finally, the focused signal light was received directly by another Golay cell detector (GC-1P, Tydex Ltd.). The experimental temperature was kept at room temperature (23°C).

### 2.2 Principles

Figure 1(b) gives the schematic diagram of the horizontally scanning principle as the stage scans along x axis. ΔABC is a section of the prism along the y axis, where point D is the midpoint of hypotenuse AC and point $D^{\prime }$ is the incident point on the slant surface corresponded to the total reflected beam at almost close to point B. The solid yellow, red, and purple lines indicate THz-wave propagation paths in prism at different location. The THz wave incident angle (defined in air) to the prism bottom is α; in other words, it is the angle between the incident surface of THz-wave and z-y surface in case the prism bottom is the horizontal plane, i.e., 30 deg here. δ and θ are the incident angle at the slant surface and the incident angle at the sampling surface, respectively. β and γ are the complementary angle of the incident angle θ and the base angle of prism, respectively.

The effective imaging area is a key factor for an imaging system. In our study, we adopted a horizontal-scanning method and an isosceles triangle-shaped silicon prism with base angle γ of 49 deg. The image of a sample on the prism bottom in the x-y plane can be obtained through prism scanning, and the angle α does not change in the imaging process. According to the Snell formula, the complementary angle β is 46.5 deg and less than the base angle of prism. Thus, for the x axis image, the THz-wave can be totally reflected from points A to B at the prism bottom, as shown in Fig. 1(b). For the y axis image, the prism is moved along the same axis. In other words, this ATR prism can ensure the entire surface of prism bottom was imaged.

It is noteworthy that the effective imaging area is affected by not only the ATR prism structure but also the size of off-axis parabolic mirror 2 used to receive signal light. Figure 1(c) shows the schematic diagram of off-axis parabolic mirror 2 receiving THz signal. Points F, G, H, and I are the vertex of the projection of the off-axis parabolic mirror 2 along y axis. d is the diameter of the off-axis parabolic mirror 2. The angle $\varepsilon_{1}$ between the outgoing beam direction and the slant surface is 71 deg. The outgoing beam is received by the arc FQG (note: the line FG is used instead of arc FQG for convenience in this study). The angle $\varepsilon_{2}$ between line FG and line GH is 77.5 deg. Line segments FM and GJ are used to indicate the radius of outgoing beam (① and ②) emitted from points C and B, respectively. Outgoing beam emitted in the range of B and C can be received by the parabolic mirror. Making line segment CN parallel and equal to line segment MJ and the angle $\varepsilon_{3}$ between line CN and line segment BJ is 107.5 deg. According to the relationship between side and angle of the triangle, CN=BC $\sin\;\varepsilon_{1}/\sin\;\varepsilon_{3}$ in ΔABC. Here, the length of the hypotenuse of ATR prism is 26.5 mm, thus, the length of line segment CN is 26.27 mm. In order to ensure that the entire surface of prism bottom was imaged, the length of line segment FG should be greater than the length sum of the line segments FM, MJ, and JG, which is equivalent to 46.27 mm in case that the THz spot size is about 20 mm. The off-axis parabolic mirror with diameter of 50.8 mm was chosen in the experiment, which is larger than the THz spot size at the receiving surface of THz-wave. Therefore, all the sample information on the prism bottom can be received by off-axis parabolic mirror 2 without any loss.

**Fig. 2:**
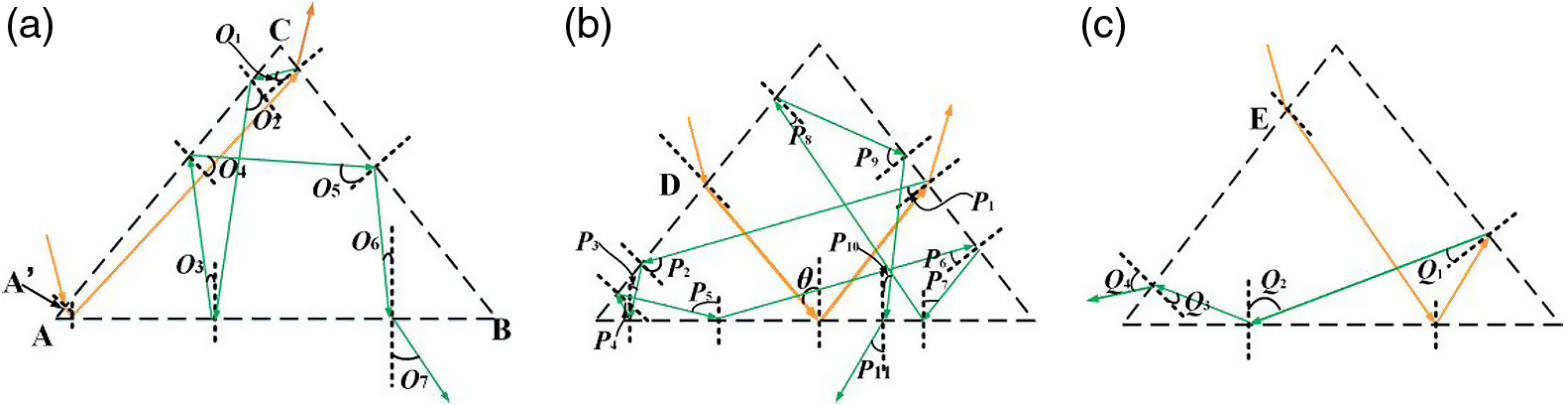
The schematic diagram of the secondary reflection effect, when the THz-wave is incident at the points (a) $A^{\prime }$, (b) D, and (c) E of the slant surface, respectively.

The light path diagram of THz-wave secondary reflection is shown in Fig. 2. The position of point $A^{\prime }$ is almost close to point A, and the position of point E is located between points D and $D^{\prime }$. The solid yellow and green lines indicate the propagation paths of THz-wave in single reflection and secondary reflection, respectively. When the THz-wave is incident at different positions of the slant surface, $O_{1}-O_{6}$, $P_{1}-P_{10}$, and $Q_{1}-Q_{3}$ are the reflection angles of THz-wave secondary reflection at the prism surface, whereas $O_{7}$, $P_{11}$, and $Q_{4}$ are the refraction angle of the secondary reflection outgoing beam. When the THz-wave is incident at almost close to point A, the reflection angles of $O_{1}-O_{6}$ are 5.5 deg, 76.5 deg, 27.5 deg, 21.5 deg, 60.5 deg, and 11.5 deg, respectively. When the THz-wave is incident at point D, the reflection angles of $P_{1}-P_{10}$ are 5.5 deg, 76.5 deg, 27.5 deg, 21.5 deg, 70.5 deg, 21.5 deg, 27.5 deg, 21.5 deg, 60.5 deg, and 11.5 deg, respectively. When the THz-wave is incident at point E, the reflection angles of $Q_{1}-Q_{3}$ are 5.5 deg, 54.5 deg, and 5.5 deg, respectively. The critical angle at the prism–air interface is about 17 deg. Therefore, after 6, 8, and 1 total reflections in Figs. 2(a)–2(c), the secondary reflection beam goes out from prism with refraction angles ($O_{7}$, $P_{11}$, and $Q_{4}$) of 43 deg, 43 deg, and 19 deg, respectively. When the incident point of THz-wave is selected in another position, the propagation path of THz-wave is similar to the cases listed above. The outgoing surfaces of the secondary reflection beam are all different from that of single reflection, thus, the imaging result will not be affected by the secondary reflection beam.

### 2.3 Sample Preparation

To verify the large effective imaging area and high sensitivity of this imaging system, we adopted rat glioma model with large size and orthotopic glioma model of nude mice with small size, respectively. The five C6-rat brain tumor models and 16 U87-MG mice brain tumor models were provided by the Third Military Medical University. The C6-glioma model was established by implanting C6 glioma cells into 3-week-old male Sprague-Dawley rats. U87-MG glioma model has a clear boundary between normal and tumor tissues. The mice samples were divided into four groups, and the number of the sample with glioma in each group was 4. For the comparison, the same surgical procedures were also performed on the normal mice. The mice glioma model was established by implanting U87-MG glioma cells with different cell densities into 5-week-old male mice. The cell density of injection into mice in each group was 0, $1.25\times 10^{6}/\mathrm{ml}$, $1.6\times 10^{6}/\mathrm{ml}$, $4\times 10^{6}/\mathrm{ml}$, respectively. The mice and rat were all allowed to grow for 2 to 3 weeks, and then the brains were extracted after euthanasia. The extracted whole brains were divided into two parts on the coronal surface with a scalpel. One part was for THz imaging and the other part was quickly put to −15°C slicing machine for staining. Considering the tissue surface used for THz imaging is in close proximity to the tissue surface used for H&E staining, we compared the results of these two parts. All animal experiments were performed in accordance with the China Animal Welfare Legislation and were approved by the Third Military Medical University Committee on Ethics for the Care and Use of Laboratory Animals.

### 2.4 Data Analysis

In order to reduce the image noises from slight power fluctuations, the reflected THz-wave beam by beam splitter and the transmitted THz-wave beam were used as the reference $I_{\text{substrate}}$ and the signal $I_{\text{sample}}$, respectively. 2-D image of sample was obtained with the pixel value of $I_{\text{sample}}/I_{\text{substrate}}$. THz imaging with and without sample were measured as the images of $F_{\text{sample}}$ and $F_{\text{substrate}}$, respectively. Considering the uniformity of the silicon prism, the reflectivity R of sample can be calculated by dividing the pixel values in $F_{\text{substrate}}$ by $F_{\text{sample}}$.

## 3 Results and Discussion

**Fig. 3:**
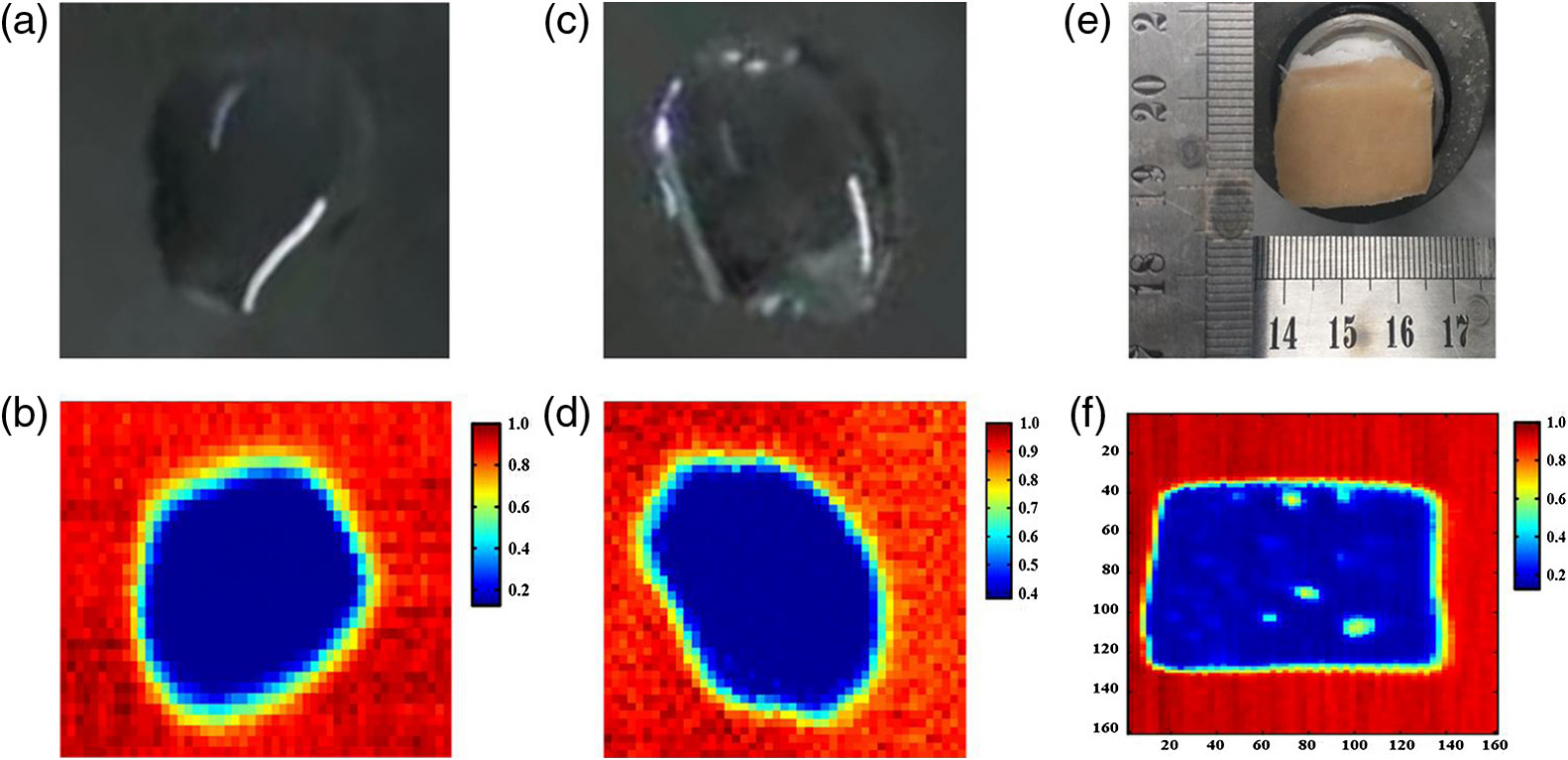
(a), (c) Visual images of a drop of water. (b), (d) THz-ATR imaging results of water under p- and s-polarizations, respectively. (e) Visual and (f) THz images of chicken under p-polarizations, respectively.

In order to verify the ATR image will not be affected by the secondary reflection beam, the experiment was carried out by imaging a drop of water under different polarizations. Figure 3(a) shows the visual image of water. Figure 3(b) is the ATR image of water with p-polarized THz-wave using a wire grid polarizer. Considering the water evaporation between two times measurements, a new water droplet shown in Fig. 3(c) was used for the THz image. Figure 3(d) indicates the THz image of a new water droplet using the s-polarized THz-wave. The color bar on the right side indicates the reflectivity (R), where a lower THz reflectivity means the larger sample absorption. The high absorption regions of THz-wave are the droplet region, as the blue regions shown in Figs. 3(b) and 3(d). It is seen that THz imaging results corresponded well with visual images. This indicates the THz imaging of water droplets were both not affected by the secondary reflection. Furthermore, a chicken sample with area of $28\times 18\;{\mathrm{mm}}^{2}$ was measured to demonstrate the large effective imaging area, whereas the area of prism bottom is $32\times 32\;{\mathrm{mm}}^{2}$, as shown in Fig. 3(e). Figure 3(f) shows the THz image for chicken tissue. The pixel numbers of chicken and background areas are 140×90=12,600 and 160×160=25,600, respectively. The measurement times were ∼21  min. The size of the chicken tissue shown in the THz image was same to that of the corresponding visual image. This indicates that this ATR imaging system can realize large effective imaging area.

**Fig. 4:**
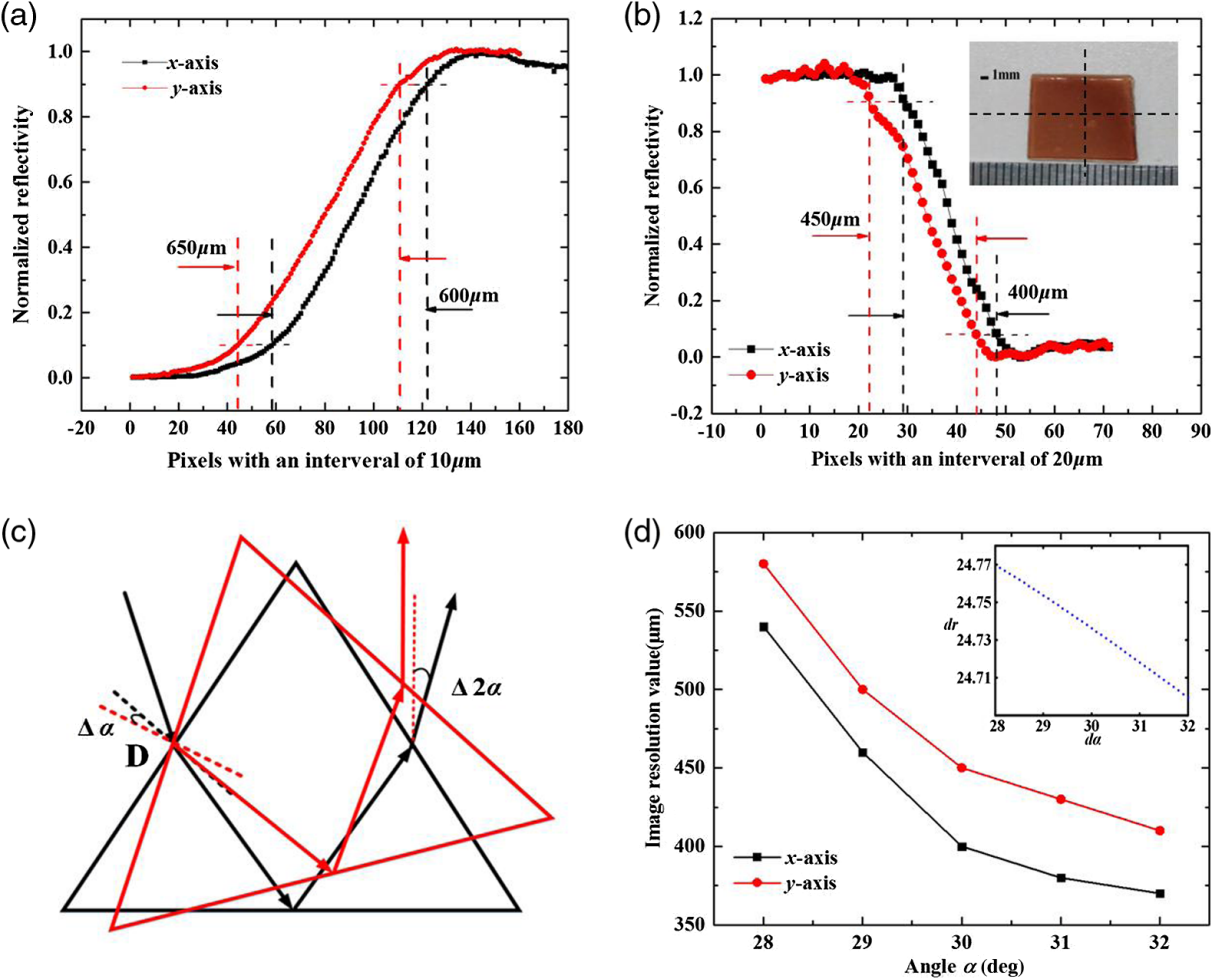
(a) Measurement of the focal spot size (without prism) by the knife-edge method. (b) The x and y diameters of imaging resolution (with prism) by scanning across the edge of an agar at the incident angle α of 30 deg, respectively. (c) The prism rotating about point D and (d) the image resolution versus the angle α.

The spatial resolution is a key parameter of the imaging system. It was evaluated through detecting the THz focusing spot size at sample plane (without prism) using the knife-edge method. The x and y diameters measured as the 10% and 90% distance between the peak and bottom were found to be 600 and 650  μm, respectively, as shown in Fig. 4(a). However, when using ATR prism, the focused beam size will be changed due to its closely related to Rayleigh criterion.^18^ For Rayleigh criterion (r=0.61λ/NA), numerical aperture (NA=n sin θ) is proportional to the refractive index n of the medium and incident angle θ at the sampling surface. Hence, there will be a decrease of focused beam size as the refractive index of prism is greater than that of air. A piece of rectangular blood agar placed at the center of prism bottom is measured, through which the spatial resolution of this imaging system is estimated. Figure 4(b) shows two lines scanning across the edge of an agar with step size of 20  μm, which were marked by the black dotted line in the inset of Fig. 4(b). Based on the 10%−90% criterion of the normalized reflectivity, the image resolution was obtained. The x and y diameters of image resolution (with prism) of this system can be reached about 400 and 450  μm, respectively. In addition, considering the incident angle θ is proportional to the angle α, the effect of different angles α on imaging resolution was studied. Figure 4(c) shows the prism rotated about point D undergoing counterclockwise and clockwise deflection about 2 deg, respectively. In this case, the light can be completely received by the THz detector. The image resolution at different angle α was achieved by scanning across the edge of an agar with step size of 20  μm, as shown in Fig. 4(d). The image resolution value decreases with the angle α increase. The relation between the Rayleigh criterion variation dr and the relative variation of the angle α at the sampling surface in theory was shown in the inset of Fig. 4(d). It is seen that the Rayleigh criterion increases as the angle α increases. The experiment result is in good agreement with the theoretical calculation.

**Fig. 5:**
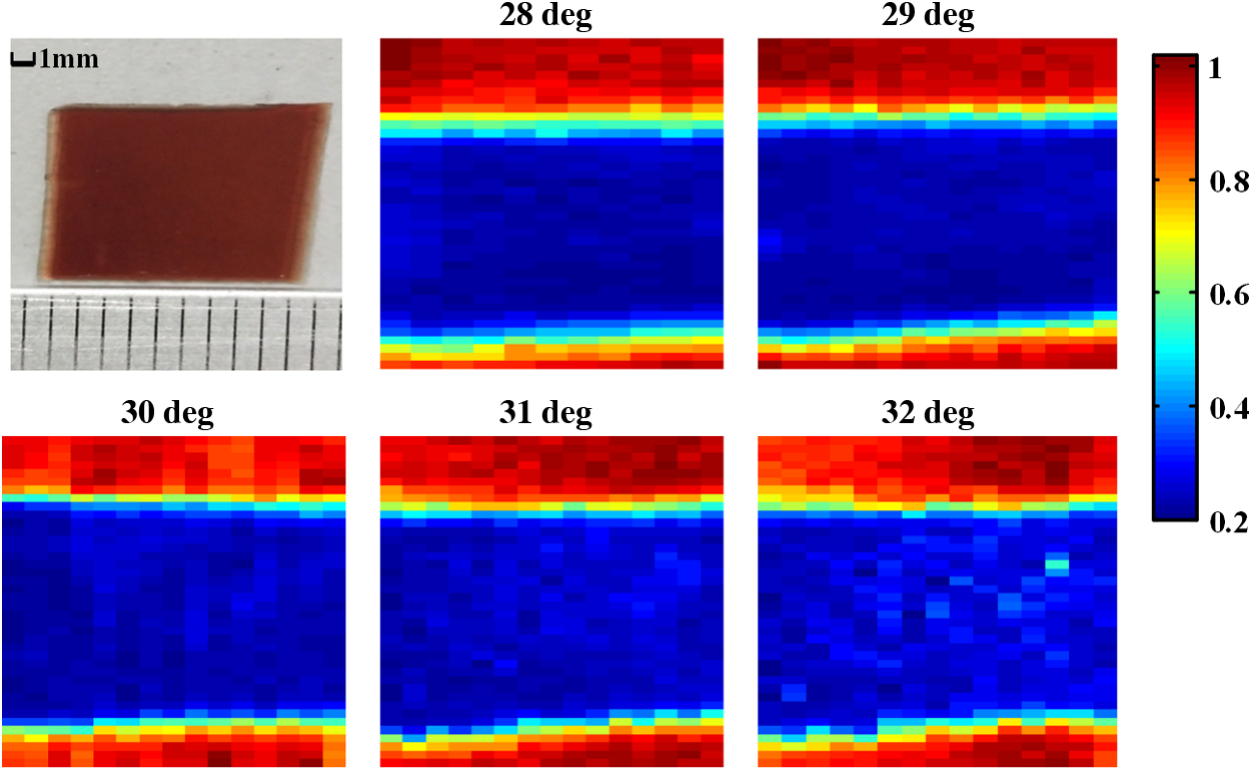
Visual and THz-ATR images of blood agar as the function of the angle α.

Then, we compared the reflectivity changes under different angles α. Figure 5 shows the THz ATR imaging of blood agar with different angles α. The blood agar regions displayed as THz-wave high absorption area can be differentiated clearly from the background. The average THz reflectance values for the blood agar regions are 26.5%, 27%, 27.8%, 28.6%, and 32.5% at angle α of 28 deg to 32 deg, respectively. Compared to the angle α of 30 deg, the measured results were reduced by 1.3% and 0.8% in the counterclockwise direction for 28 deg and 29 deg, and increased by 0.9% and 4.7% in the clockwise direction for 31 deg and 32 deg, respectively. For the α of 29 deg, the measured results were reduced by 0.5% for 28 deg, and increased by 0.8%, 1.6%, and 5.5% for 30 deg, 31 deg, and 32 deg, respectively. Therefore, the system with the angle α of 29 deg can have the slightly higher stability with small reflectivity change. However, taking the image resolution and stability of the horizontally scanning THz-ATR imaging system into consideration, the angle α was selected as 30 deg for achieving high resolution and stability imaging.

**Fig. 6:**
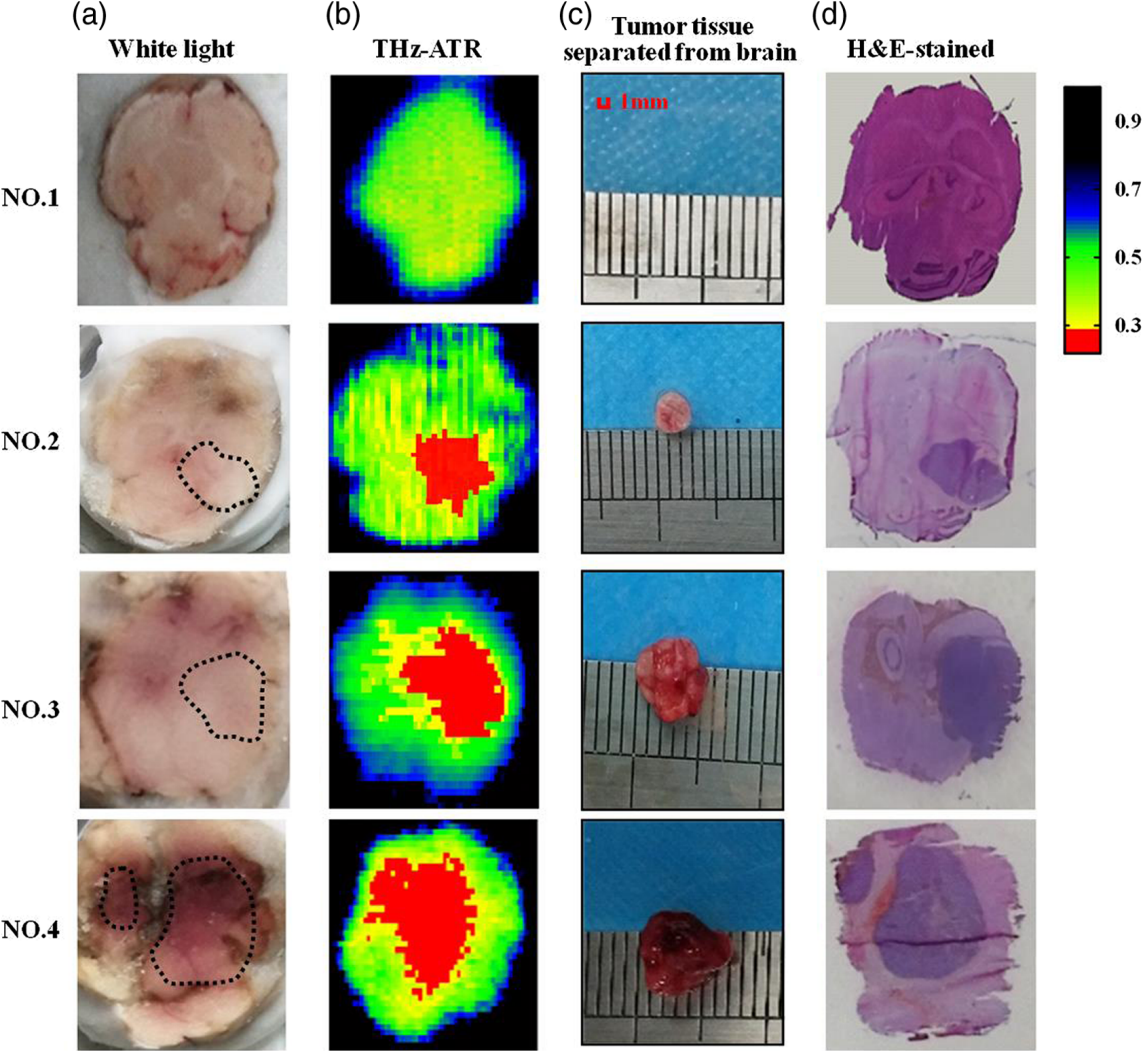
(a) Visual, (b) THz-ATR, (c) tumor tissue separated from brain, and (d) H&E-stained images of freshly excised brain tissues.

Next, THz-wave imaging was performed on freshly excised brain tissue with different tumor sizes in mice model. In our study, there are total 12 mice with glioma and 4 normal mice. Figure 6 shows the visual, THz-ATR, and H&E-stained images for brain tissues without and with tumor. There was no tumor for no. 1 sample, whereas tumor sizes of nos. 2 to 4 sample gradually increased due to the increase of implanted U87-MG glioma cell density. U87-brain tumor models have clear boundary between normal and tumor tissues, and the tumor regions in the visible images of frozen samples marked by dashed lines in Fig. 6(a). The THz-ATR imaging of one part of the extracted brains was shown in Fig. 6(b). Each pixel was described using the intensity reflectivity of the THz wave, where a lower THz reflectivity means the larger sample absorption. The tumor region could be distinguished clearly from the normal regions in the THz images, and the intensities of the THz signals in the cancerous brain tissue were lower than those for the normal brain tissue. This can be attributed to the water content in the tumor region is higher than that in normal tissue.^19^ It is clearly seen that the THz reflection image in the sham group looks uniform, with the average reflectivity of 40%. The THz images of brain tissues with different tumor size depicted obvious difference compared with sham group. To evaluate the effect of different cell injection concentrations into mice on tumor size, we separate tumor tissue from brain tissue after THz-ATR image. Figure 6(c) shows the images of tumor size in each group. It can be seen that the size of tumor increased with the concentration of injected cells into mice. In order to verify the accuracy of THz-ATR image, the glioma region of the other part of the extracted brain tissues was pathologically determined with the H&E-stained image, as shown in Fig. 6(d). The tumor region is recognized as an area with deep purple hematoxylin staining, corresponding that the cell nuclei density of tumor areas is higher than that of normal tissues.^10^ The tumor regions in THz images are similar to those of the corresponding visual and H&E-stained images. Furthermore, we estimated image size of the tumor detected by THz-ATR, visual (tumor tissue separated from mice brain), and H&E staining for nos. 2 to 4 samples, as listed in Table 1. The size of abnormal regions indicated clearly by THz image was larger than those detected using H&E-stained and visual images. There are two main reasons for these results. One reason is the relatively low spatial resolution of the ATR image system. Another reason is that THz-wave is sensitive to the distribution of water content and cellular hydration state and THz imaging could indicate the presence of perifocal edema around the tumor region.^20^ Considering the high sensitivity of this horizontal-scanning THz-ATR imaging system, the identification of tumor region with smaller size can be facilitated by further improving the image resolution.

**Table 1:** Tumor sizes of THz-ATR and histological images.

	Tumor sizes (${\mathrm{mm}}^{2}$)		
Mice no.	THz-ATR	Tumor tissue separated from brain	H&E-stained image
2	9.05	8.80	8.30
3	25.20	24.50	22.50
4	50.34	48.50	46.60

**Fig. 7:**
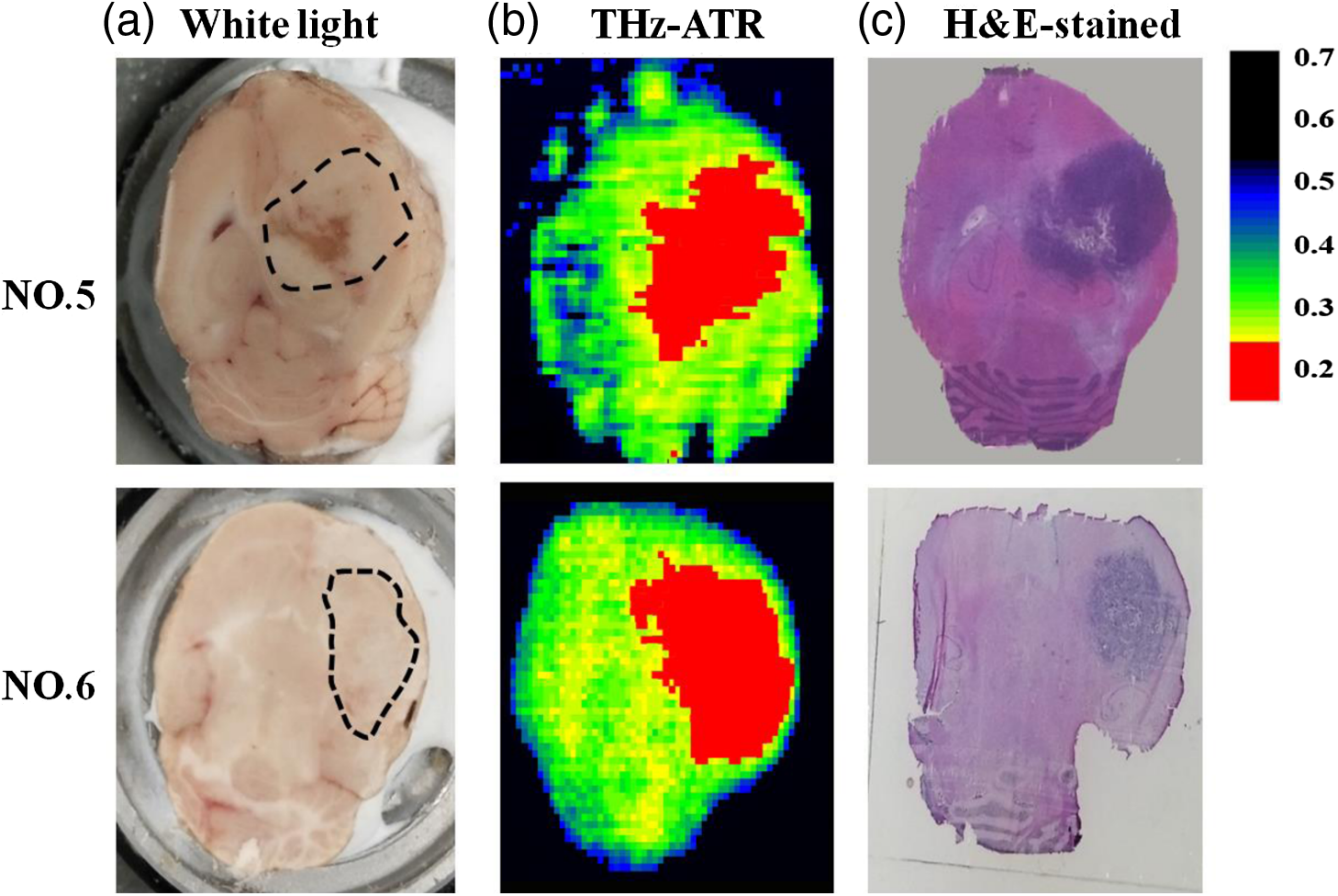
(a) Visual, (b) THz-ATR, and (c) H&E-staining images of freshly excised brain tissues.

The biomedical application for large area sample of the THz ATR imaging system was demonstrated by freshly excised brain glioma tissues in rat model. Figure 7 shows the visual, THz-ATR, and H&E-staining images of brain glioma in rat model. The tumor regions were marked by the dotted line, indicated by Fig. 7(a). The scanning areas of glioma samples (no. 5 and no. 6) are about $12\times 20\;{\mathrm{mm}}^{2}$ and $12\times 15\;{\mathrm{mm}}^{2}$, respectively. Figure 7(b) shows the THz-ATR images of one part of the extracted rat brain tissues, and the THz high absorption regions are tumor regions. The dark purple regions in H&E-stained images are tumor regions, as shown in Fig. 7(c). In general, the tumor locations and regions of THz images are consistent with those in H&E-stained and visual images. However, the process of tumor formation is different between humans and model rats. Especially, human tumors are generated invasively, whereas tumors in model rats agglutinate as a cluster. Therefore, further investigations with human tissue are necessary to evaluate the practicability of this method in clinical surgery.

## 4 Conclusions

We set up a horizontal scanning CW THz-ATR imaging with a large effective imaging area. The image resolution and stability of this proposed scanning method have been analyzed in detail. The experimental results show that this system has high image resolution and stability at α=30  deg. The C6-glioma regions of fresh rat brain tissues and U87-glioma regions with different sizes of mice brain tissues all can be well distinguished by THz imaging, and were in agreement with visual and H&E-stained images. The identification of smaller sized tumor tissue can also be facilitated by further improving the image resolution of THz ATR imaging systems, by using the prism with higher refractive index material and adopting the higher incident angle α of the THz wave. Therefore, based on the high sensitivity and nondestructiveness of THz-ATR imaging, it bears great potential to be applied for *in situ* and *in vivo* measurements of biological samples.
